# Crystal recombination control by using Ce doped in mesoporous TiO_2_ for efficient perovskite solar cells†

**DOI:** 10.1039/c8ra07800a

**Published:** 2019-01-09

**Authors:** Honglin Lu, Jia Zhuang, Zhu Ma, Weiya Zhou, Haoran Xia, Zheng Xiao, Hua Zhang, Haimin Li

**Affiliations:** The Center of New Energy Materials and Technology, School of Materials Science and Engineering, Southwest Petroleum University Chengdu 610500 P. R. China zj-656@163.com deve198509@163.com +86 02883033286 +86 13550396098 +86 13880863057

## Abstract

Efficient electron transport layers (ETLs) are the crucial issue for electron transport and hole blocking in organic–inorganic hybrid perovskite solar cells (PSCs). To date, most of the reported effective ETLs have comprised TiO_2_, which exhibits limited electron mobility and numerous defect states and restricts the enhancement of the performance of PSCs. Hence, the investigation of effective tactics for improving the electronic properties of TiO_2_ is critical for the fabrication of high-efficiency devices. In this study, a cerium doping method was adopted in mesoporous TiO_2_, which was prepared *via* a traditional one-step hydrothermal process, to improve its electron transport properties by recombining nanocrystals and optimizing the negative flat band potential of TiO_2_. Continuous, aligned and regulated recombined crystals of mesoporous TiO_2_ were obtained with optimized pathways of electron transport from the ETL to the FTO layer. Moreover, a small amount of Ti^4+^ ions was replaced by Ce^4+^ ions in the TiO_2_ lattice, which led to deformation of the TiO_2_ lattice and influenced the growth process of TiO_2_ grains. With an optimized mole proportion of Ce element in the TiO_2_ precursor, the power conversion efficiency (PCE) of perovskite solar cells was typically boosted to 17.75% in comparison with 15.92% in the case of undoped TiO_2_.

## Abstract 2

*J*–*V* curves of PSCs based on TiO_2_ and 2% Ce-TiO_2_ ETLs.

## Graphical abstract

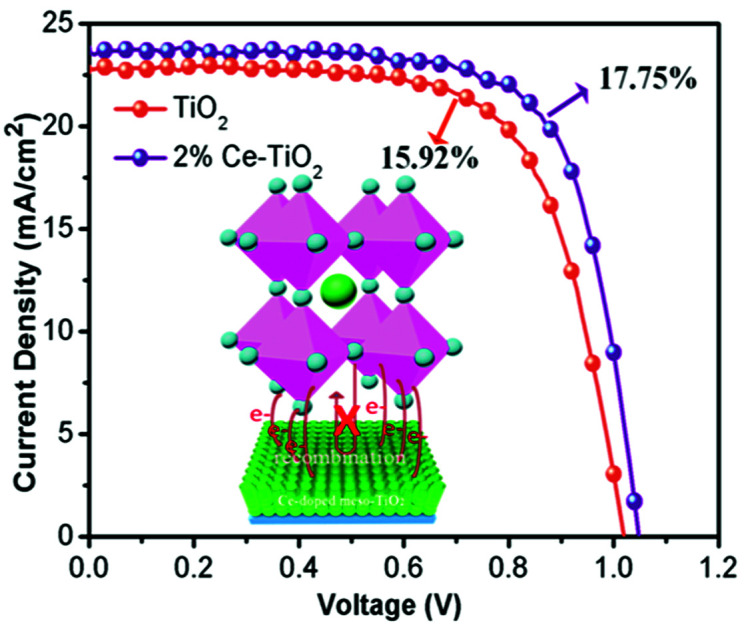

## Introduction

In recent years, numerous works have focused on organic–inorganic hybrid perovskite solar cells (PSCs) owing to their strong photovoltaic properties and low cost of manufacture. Their photoelectric conversion efficiency (PCE) has increased enormously from 3.8% in 2009 to 23.3% in 2018.^1–3^ Owing to the untiring efforts of researchers, PSCs will have great potential as replacements for silicon-based solar cells. In typical PSCs, a perovskite absorber layer, either with or without a mesoporous scaffold, is sandwiched between the electron transport layer (ETL) and the hole transport layer (HTL).^4,5^ The ETL has a significant function in transporting electrons and blocking holes.^6–8^

To date, various metal oxide films have been employed as ETLs, such as TiO_2_, ZnO, SnO_2_, CeO_2_, BaSnO_3_, Nb_2_O_5_, SrTiO_3_ and In_2_S_3_,^9–17^ of which TiO_2_ has been the most frequently used material. In comparison with compact TiO_2_ (c-TiO_2_), mesoporous TiO_2_ (m-TiO_2_) is usually used as a scaffold over c-TiO_2_ to enhance the electron transport, light absorption and environmental stability of PSCs.^18,19^ At present, most high-efficiency PSCs are based on m-TiO_2_. However, m-TiO_2_ suffers from many problems, such as the surface and internal oxygen vacancies in m-TiO_2_, low conductivity, and multiple defect state densities, which lead to the loss of performance in PSCs.^20,21^

Surface passivation is considered to be an efficient method of boosting electron transport in silicon-based solar cells and PSCs from the absorption layer to the electrodes or ETLs and reducing electron recombination, which further increases the PCE of the device. Many groups have focused on the interfacial engineering of ETLs to increase the PCE of cells. For instance, Jia *et al.* introduced CsI as an interfacial modification layer between MAPbI_3_ and m-TiO_2_, which could improve the surface morphology of m-TiO_2_, reduce the work function of TiO_2_, and reduce the surface defect density.^22^ Zheng *et al.* reported an easy strategy whereby the deposition of both aminocaproic acid and caproic acid between mesoporous TiO_2_ and an MAPbI_3_ layer achieved a significant increase in PCE, which could contribute to the accelerated extraction and transfer of electrons *via* interfacial modification.^23^ Although the surface passivation of m-TiO_2_ can notably increase the PCE of devices, this additional modification process is contrary to the requirement for a simple fabrication process for the industrialization of PSCs. Therefore, it is important to search for a cost-effective and easy manufacturing method for processing high-quality and efficient m-TiO_2_ ETLs.

Recently, an ion doping method has been used to replace this additional modification process and moreover improve the optoelectronic properties of m-TiO_2_ ETLs. At present, many researchers have reported the ion doping of ETLs to increase the PCE of devices. For example, in the case of tungsten-doped TiO_2_ it was demonstrated that the conduction band (CB) of TiO_2_ was shifted downward, which is beneficial for electron transport because of the optimized energy level matching between the lowest unoccupied molecular orbital (LUMO) of an MAPbI_3_ layer and the CB of TiO_2_.^24^ Su *et al.* demonstrated that niobium (Nb)-doped TiO_2_ was manufactured *via* a one-step spin-coating process and the PCE of a cell increased from 14.9% to 16.3%, which was attributed to the increased charge density and conductivity of the TiO_2_ ETL.^25^ Chen *et al.* reported that in PSCs based on lithium-doped TiO_2_ the density of electron trap states can be increased.^26^ Moreover, aluminum, cesium and cadmium have been doped into TiO_2_ ETLs to improve the performance of PSCs by controlling the carrier dynamics in TiO_2_.^27–29^ Thus far, the aim of doping ions into TiO_2_ has mainly been to enhance the electronic properties of ETLs, which is mainly attributed to the high conductivity of the doped metal itself and the improved alignment of energy levels. However, few researchers have proposed schemes for the improvement of electronic properties by optimizing the morphology of TiO_2_ films by ion doping, which promotes electron transport in ETLs and decreases electron recombination.

In this study, a crystal recombination phenomenon was observed after cerium element was doped into mesoporous TiO_2_, which was prepared using a one-step hydrothermal method. The regulated crystal recombination provided optimized transport pathways for the extraction and transport of electrons. PSCs exhibited a maximum PCE of 17.75% with an increased open-circuit voltage (*V*_OC_), short-circuit current density (*J*_SC_), and fill factor (FF) in comparison with undoped cells. The overall enhancement in performance was ascribed to the excellent electronic properties of the ETL.

## Results and discussion

**Fig. 1:**
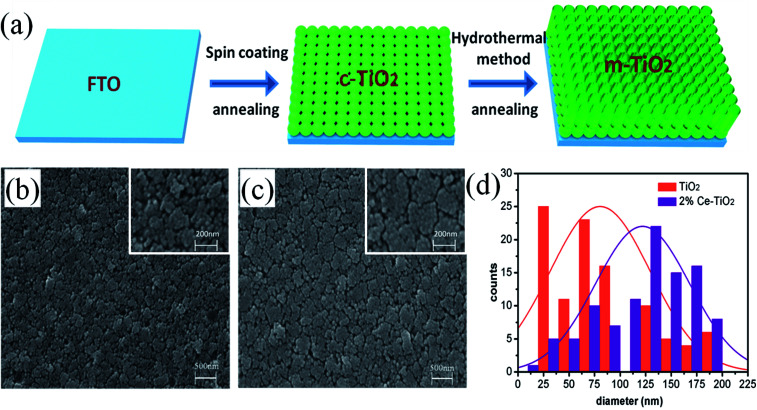
(a) Schematic flow chart of the fabrication of compact TiO_2_ and mesoporous TiO_2_, (b) and (c) SEM images of FTO/c-TiO_2_/m-TiO_2_ and FTO/c-TiO_2_/2% Ce-doped m-TiO_2_, (d) diameters of crystalline grain agglomerates of mesoporous TiO_2_.

Fig. 1a shows a schematic flow chart of the fabrication process of mesoporous TiO_2_. Compact TiO_2_ was prepared by spin coating, and mesoporous TiO_2_ was fabricated by a hydrothermal method. SEM images of mesoporous TiO_2_ and mesoporous TiO_2_ doped with 2% Ce are presented in Fig. 1b and c, respectively. As seen in Fig. 1b and c, we obtained nanoparticle-shaped TiO_2_ by a hydrothermal method, whereby mesoporous TiO_2_ doped with 2% Ce exhibited recombined crystalline grains with a greater diameter and a smoother nanodruse surface than undoped m-TiO_2_. The morphology of Ce-doped m-TiO_2_ promoted the transport and penetration of electrons from the MAPbI_3_ absorption layer into the mesoporous ETL, which possessed planar and mesoporous characteristics.^30,31^Fig. 1d shows the diameter distribution of crystalline grain agglomerates, which further demonstrates that the diameter of crystalline grain agglomerates was larger when 2% Ce ions were doped into mesoporous TiO_2_.

**Fig. 2:**
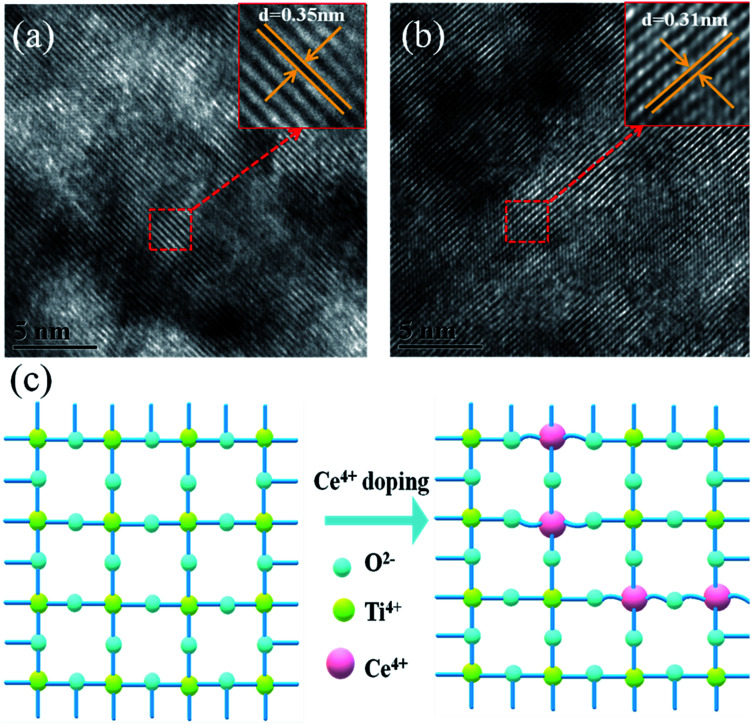
HRTEM images of (a) TiO_2_ and (b) 2% Ce–TiO_2_ and (c) 3D model diagram of Ce^4+^ ions replacing Ti^4+^ ions in the TiO_2_ lattice.

High-resolution transmission electron microscopy (HRTEM) was used to elucidate the reason for the lattice deformation that occurred when 2% Ce was doped into m-TiO_2_ (Fig. 2a and b). The lattice spacing measured from an HRTEM image was 0.35 nm (inset of Fig. 2a), which corresponded to the (101) crystallographic planes of the anatase form of titanium dioxide. Doping 2% Ce into TiO_2_ led to lattice deformation and a decrease in the spacing (0.31 nm) between the (101) crystallographic planes of the anatase form of titanium dioxide. This result shows that Ce element penetrated into the TiO_2_ lattice. Because the ionic radius of Ce^4+^ ions is greater than that of Ti^4+^ ions, the extrusion effect of ion replacement caused the lattice spacing to decrease. Fig. 2c illustrates the reason why mesoporous TiO_2_ doped with 2% Ce had a greater diameter of crystalline grain agglomerates when Ce^4+^ ions were doped into the TiO_2_ lattice.^32,33^ (A study showed that the critical temperature for the valence state from Ce^3+^ ion to Ce^4+^ ion is 200 °C. The experimental temperature was 500 °C, indicating that the conversion to valence state is credible.) Ce atoms have higher bond energies than Ti atoms, which could enable substitution doping when Ce^4+^ ions were doped into the TiO_2_ lattice.^32^ The incomplete match between the geometric features of the two elements led to slight deformation and strain in the TiO_2_ lattice. The greater diameter of recombined crystalline grains of TiO_2_ nanoparticles that was observed can be attributed to the occurrence of lattice deformation and strain induced by the substitution of larger Ce^4+^ ions (94 pm) in the positions of Ti^4+^ ions (68 pm). Lattice deformation caused by substitution doping leads to larger crystal particles, improves the regularity of the crystal structure, decreases the number of irregular holes, and provides more channels and opportunities for electron transport. Frank *et al.* reported that a decrease in film porosity was beneficial for electron transport in an m-TiO_2_ film.^34^ High porosity implies longer electron transport pathways, which indicates that electrons will spend a longer time in the m-TiO_2_ network before being collected.^34,35^ This result will cause more electron recombination. m-TiO_2_ doped with 2% Ce (Fig. 1c) exhibited lower porosity than undoped m-TiO_2_ (Fig. 1b), which was beneficial for improved electron transport and further increased the PCE of cells.

**Fig. 3:**
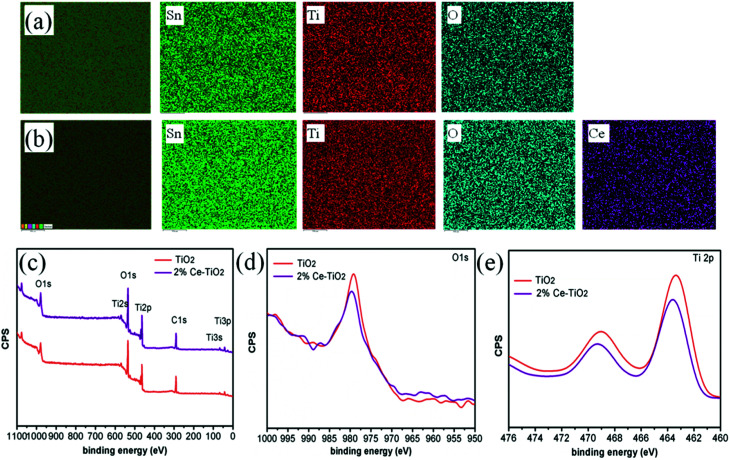
EDX elemental images of (a) FTO/c-TiO_2_/m-TiO_2_ and (b) FTO/c-TiO_2_/2% Ce-doped m-TiO_2_. XPS measurements of TiO_2_ and Ce-doped TiO_2_ mesoporous ETLs: (c) survey spectrum, (d) O 1s spectrum, and (e) Ti 2p spectrum.

Fig. 3a and b show elemental maps of the elemental compositions of undoped and 2% Ce-doped mesoporous TiO_2_ ETLs, respectively. Tin element originated from FTO glass, and Ti element originated from c-TiO_2_ and m-TiO_2_. The presence of cerium element and its uniform distribution demonstrate that Ce was successfully doped into mesoporous TiO_2_ by doping cerium nitrate pentahydrate into the hydrothermal precursor solution.^36^ To further confirm the presence and intrinsic effect of cerium element in TiO_2_, XPS (the carbon peak correction was carried out) was used to determine the chemical compositions and chemical states of undoped mesoporous TiO_2_ and mesoporous TiO_2_ doped with 2% Ce.^37^ The full XPS spectra can be seen in Fig. 3c, which shows the presence of Ti, O, and C elements in undoped mesoporous TiO_2_ and mesoporous TiO_2_ doped with 2% Ce. The presence of a C 1s peak is ascribed to adsorbed hydrocarbons on the TiO_2_ surface. However, the presence of Ce element is not directly indicated in Fig. 3c owing to its low concentration, but its characteristic peak can influence shifts in other peaks of mesoporous TiO_2_, which could confirm the presence of Ce indirectly. Fig. 3d shows the O 1s peaks of undoped mesoporous TiO_2_ and mesoporous TiO_2_ doped with 2% Ce. The Ti 2p spectra in Fig. 3e show that mesoporous TiO_2_ doped with 2% Ce exhibited a peak shift from 463.4 eV to 463.6 eV in comparison with undoped mesoporous TiO_2_, *i.e.*, a shift of 0.2 eV. The shifts in the peaks of Ti 2p and O 1s are assigned to the different coordination environments and chemical environments that resulted when Ce^4+^ ions were doped into mesoporous TiO_2_. The shifts in the peaks of O 1s and Ti 2p are ascribed to the changes in the charge densities of O and Ti atoms, which could be caused by electron transfer from O 1s orbitals and the 2p orbitals of Ti atoms to Ce 4f orbitals. This result influenced the nucleation and crystallization of m-TiO_2_ when Ce was doped into m-TiO_2_, which further improved the morphology of m-TiO_2_. The characterization *via* EDS and XPS shows that Ce element was successfully doped into mesoporous TiO_2_ and was uniformly distributed.

**Fig. 4:**
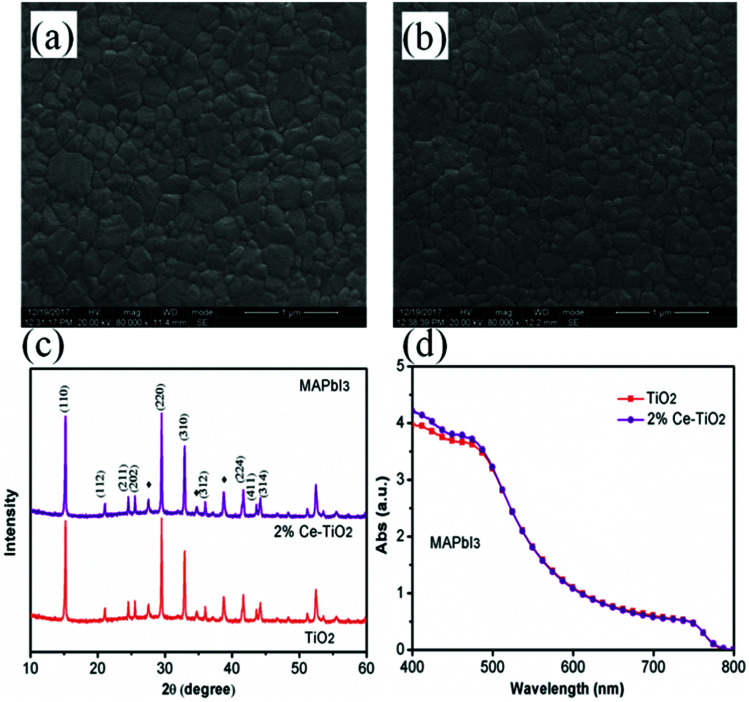
(a) and (b) Top-view SEM images of MAPbI_3_ films based on mesoporous TiO_2_ and 2% Ce-doped mesoporous TiO_2_ ETLs, (c) XRD patterns of MAPbI_3_ films (♦ represents FTO), and (d) UV-vis absorption spectra of MAPbI_3_ films based on mesoporous TiO_2_ and 2% Ce-doped mesoporous TiO_2_ ETLs.

Surface SEM images of perovskite films based on undoped mesoporous TiO_2_ and mesoporous TiO_2_ doped with 2% Ce are shown in Fig. 4a and b, respectively. When Ce was added to mesoporous TiO_2_, there was no observable change in the morphology of the perovskite film in comparison with pure mesoporous TiO_2_. This result shows that the decrease in porosity when Ce was doped into m-TiO_2_ had no influence on the morphology of the perovskite layer. The perovskite films deposited onto mesoporous TiO_2_ and Ce-doped mesoporous TiO_2_ ETL substrates exhibited uniform film-forming abilities, which is important for producing high-efficiency PSCs. Fig. 4c shows the XRD patterns of perovskite thin films deposited on mesoporous TiO_2_ and 2% Ce-doped mesoporous TiO_2_ ETLs, which demonstrate that the addition of cerium nitrate did not influence the crystal structure of MAPbI_3_ and a perovskite film structure was formed. UV-vis absorption spectra (Fig. 4d) show that the CH_3_NH_3_PbI_3_ film based on a 2% Ce-doped TiO_2_ ETL exhibited a stronger absorption peak due to the CH_3_NH_3_PbI_3_ film within the range of 400–450 nm than the CH_3_NH_3_PbI_3_ film based on an undoped TiO_2_ ETL, and hence the absorption of visible light by the perovskite layer was further improved. This result further shows the improvement in the PCE of cells after Ce was doped into m-TiO_2_.

**Fig. 5:**
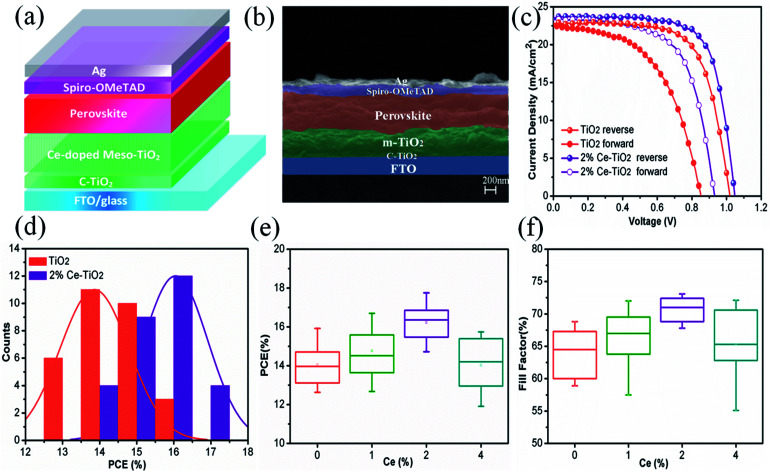
(a) Cell model diagram, (b) cross-sectional SEM image of PSC (FTO/compact TiO_2_/mesoporous TiO_2_/CH_3_NH_3_PbI_3_/spiro-OMeTAD/Ag), (c) *J*–*V* curves, (d) histogram of PCE, and (e) and (f) error bars of PCE and FF.

We manufactured a perovskite solar cell with a cell structure of FTO/c-TiO_2_ (70 nm)/m-TiO_2_ (300 nm)/MAPbI_3_ (500 nm)/spiro-OMeTAD (150 nm)/Ag (100 nm) (Fig. 5a), and a corresponding cross-sectional SEM image is shown in Fig. 5b. The reverse and forward current–voltage (*J*–*V*) characteristics of solar cells with and without 2% Ce dopant are shown and summarized in Fig. 5c and Table 1, respectively. The undoped TiO_2_ cell exhibited a *J*_SC_ value of 22.80 mA cm^−2^, a *V*_OC_ value of 1.020 V, and an FF of 68.50%, with a PCE of 15.92% under reverse scanning (10.11% under forward scanning), whereas the 2% Ce–TiO_2_ PSC exhibited a PCE of 17.75% (*V*_OC_ = 1.048 V, *J*_SC_ = 23.61 mA cm^−2^, FF = 71.70%) under reverse scanning and a PCE of 13.81% under forward scanning. A decrease in the hysteresis index (to 0.22) was demonstrated for the cell based on 2% Ce-m-TiO_2_ as the ETL (in comparison with the hysteresis index of 0.36 for the undoped device), which can be ascribed to the improved morphology and increased conductivity of m-TiO_2_. From Table S1,† we can demonstrate that the *J*_SC_ and *V*_OC_ values and FF of Ce-doped cells increased in comparison with the undoped TiO_2_ cell when the concentration was 1% and 2%. When the dopant concentration was 4%, the *J*_SC_ and *V*_OC_ values and FF of the cell declined rapidly. Ce^4+^ ions doped into m-TiO_2_ could produce a new impurity energy level, which may help charge capture and separation and may also serve as a recombination center for carriers. Obviously, doping with 4% Ce^4+^ ions provided m-TiO_2_ with recombination centers and further exerted a passive influence on the ETL. Therefore, Ce^4+^ ions were added to increase the *J*_SC_ and *V*_OC_ values and FF, which further led to an increase in the PCE at a certain concentration. The statistical distribution of the PCEs of undoped and 2% Ce-doped devices is shown in Fig. 5d, from which the PCEs of cells based on 2% Ce–TiO_2_ were concentrated around 15% and 16% in comparison with 13% and 14% in the case of undoped devices. The error bars (Fig. 5e, f and S1†) show that the 2% Ce-doped TiO_2_ cells exhibited higher *V*_OC_ and *J*_SC_ values and FFs than the undoped devices, which further illustrates that doping with Ce was effective for improving cell performance.

**Table 1:** Photovoltaic performance of PSCs based on TiO_2_ and 2% Ce–TiO_2_ ETLs

ETL	*V*_OC_ (V)	*J*_SC_ (mA cm^−2^)	FF (%)	PCE (%)	Hysteresis index
TiO_2_ reverse	1.020	22.80	68.50	15.92	0.36
TiO_2_ forward	0.852	22.51	52.70	10.11	
2% Ce–TiO_2_ reverse	1.048	23.61	71.70	17.75	0.22
2% Ce–TiO_2_ forward	0.931	23.32	63.60	13.81	

**Fig. 6:**
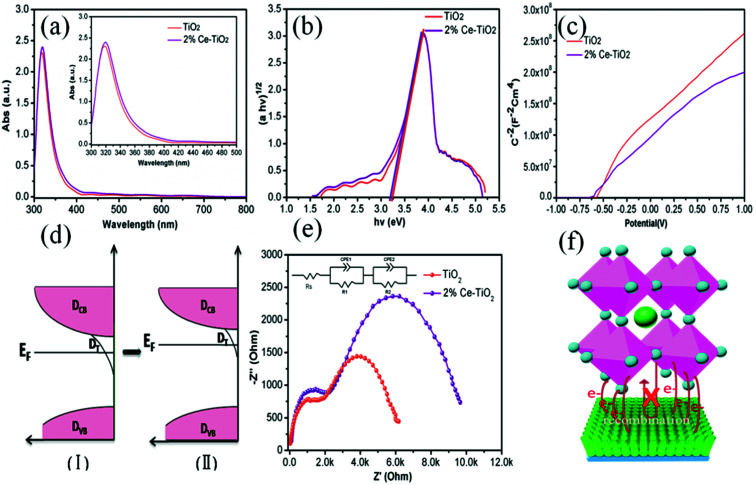
(a) UV-vis absorption spectra of TiO_2_ and 2% Ce-doped mesoporous TiO_2_ ETLs, (b) band gap map of TiO_2_, (c) Mott–Schottky curves for TiO_2_ and Ce-doped mesoporous TiO_2_ ETLs, (d) electronic density of states for meso-TiO_2_ ETLs including the densities of conduction band states (*D*_CB_), trap states (*D*_T_), and valence band states (*D*_VB_) and the decrease in the density of surface trap states due to doping with Ce, (e) EIS spectra of perovskite solar cells based on TiO_2_ and 2% Ce-doped mesoporous TiO_2_ as ETLs, and (f) 3D map of electron transport from MAPbI_3_ to the ETL.

In order to get an insight into the relationship between the performance of devices and the properties of Ce-doped mesoporous TiO_2_, different tactics were used. Fig. 6a shows the UV-vis absorption spectra of undoped and 2% Ce-doped mesoporous TiO_2_. The absorption spectrum of Ce-doped TiO_2_ displays a red shift in the band gap (small image in Fig. 6a). The transformed Kubelka–Munk spectra (Fig. 6b) of undoped and 2% Ce-doped TiO_2_ were recorded to determine the optical band gap. The optical band gap of TiO_2_ decreased from 3.25 to 3.20 eV when 2% Ce(NO_3_)_3_ was doped into the TiO_2_ precursor solution. This could be attributed to the formation of a Ti–O–Ce bond structure in Ce-doped m-TiO_2_ owing to the partial substitution of Ti by Ce, which caused the red shift in the band gap of TiO_2_. The decrease in the band gap can promote an increase in the force of charge injection from the ETL to the FTO glass layer, as shown by the increased *J*_SC_ value of the device. Fig. 6c shows the Mott–Schottky curves for undoped and 2% Ce-doped mesoporous TiO_2_. The flat band potential (*V*_fb_) is the external voltage and corresponds to the intercept with the *x*-axis. A shift in the *V*_fb_ value is related to the position of the conduction band of m-TiO_2_.^37^Fig. 6c shows that the *V*_fb_ value was negatively shifted from −0.601 V to −0.621 V, whereas the negative shift in the *V*_fb_ value implies that the quasi-Fermi level was higher (Fig. 6d). These results further led to an upward shift in the conduction band, which resulted in an increase of 15 mV in the *V*_OC_ value. The increase of 28 mV observed in the *V*_OC_ value of the device was much greater than the increase of 15 mV in the *V*_OC_ value due to the shift in the conduction band, which may be ascribed to the improved morphology and increased conductivity of m-TiO_2_. Fig. 6e shows the EIS spectra of devices based on undoped and 2% Ce-doped TiO_2_ as ETLs, which were recorded with an amplitude of 7.5 mV and a measurement frequency ranging from 1 to 100 kHz.^38,39^ The model in the inset of Fig. 6e shows the fitted equivalent circuit. Table 2 shows the values used for fitting. The arc at high frequencies is attributed to the contact resistance of the interface between the perovskite layer and the ETL, whereas that at low frequencies originated from the recombination resistance (*R*_rec_). The arc at high frequencies shows that doping with 2% Ce had little influence on electron transfer from the perovskite layer and ETL in comparison with the undoped device. The larger semicircle in the curve is generally related to the recombination of electrons. Obviously, the PSC based on mesoporous TiO_2_ doped with 2% Ce as the ETL exhibited a higher recombination resistance (8535 Ω) than the undoped cell (5420 Ω), which indicated that a lower recombination loss occurred when Ce element was doped into meso-TiO_2_. This result led to increases in the FF and *V*_OC_ value of PSCs. Fig. 6f shows a 3D map of electron transport from MAPbI_3_ to the ETL. The higher PCE of the device based on 2% Ce–TiO_2_ as the ETL decreased the recombination of electrons owing to the optimized morphology of mesoporous TiO_2_, the decreased *V*_fb_ value and the increased conductivity in comparison with the undoped device.

**Table 2:** Fitted EIS data of PSCs based on TiO_2_ and 2% Ce–TiO_2_ ETLs

Sample	Contact resistance (*R*_S_/Ω)	Transfer resistance (*R*_1_/Ω)	Transfer resistance (*R*_2_/Ω)
TiO_2_	29.37	1536	5420
2% Ce–TiO_2_	32.43	1725	8535

**Fig. 7:**
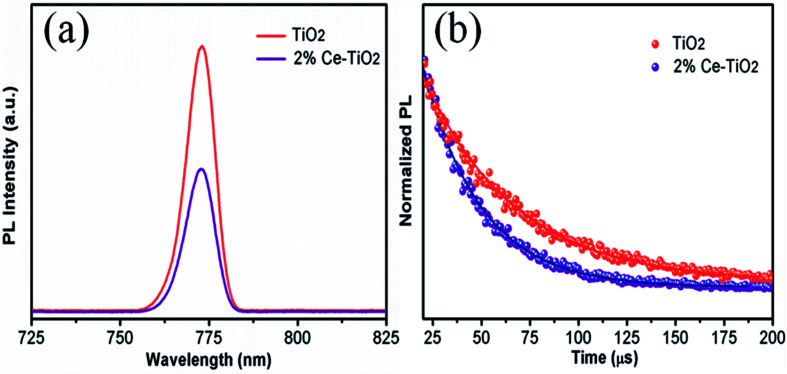
(a) PL and (b) TRPL decay curves for FTO/compact TiO_2_/mesoporous TiO_2_/MAPbI_3_ and FTO/compact TiO_2_/2% Ce-doped mesoporous TiO_2_/MAPbI_3_ films.

PL (Fig. 7a) and TRPL (Fig. 7b) measurements were used to assess whether charges could be efficiently extracted from the MAPbI_3_ layer to the mesoporous TiO_2_ layer.^40,41^Fig. 7a shows the steady-state PL spectra of PSCs with the structures FTO/compact TiO_2_/mesoporous TiO_2_/perovskite and FTO/compact TiO_2_/2% Ce-doped mesoporous TiO_2_/perovskite. From the PL peak at about 775 nm, the CH_3_NH_3_PbI_3_ perovskite film deposited on 2% Ce-doped mesoporous TiO_2_ exhibited more evident PL quenching in comparison with the pristine mesoporous TiO_2_ film, which demonstrated an enhanced charge extraction ability. Furthermore, the time constants *τ*_e_ were determined by fitting the TRPL curves (Fig. 7b) to calculate the extraction lifetimes for perovskite layers on mesoporous TiO_2_ with and without a Ce dopant as the ETL. As a result, the fitted time constants *τ*_e_ were calculated by a biexponential decay function as follows:1
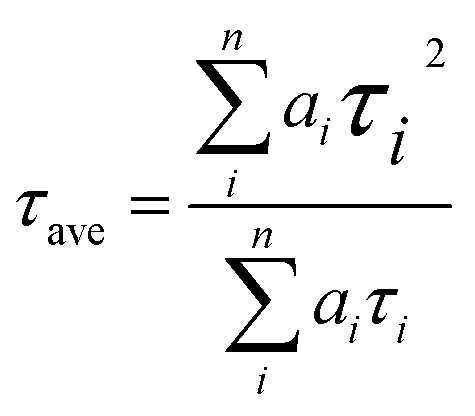


The results for the *τ*_e_ values (Table 3) were 37.40 ns for the doped cell and 51.93 ns for the undoped cell, respectively. Thus, the PL decay for the cell based on 2% Ce-doped TiO_2_ was approximately 27.98% faster in comparison with the PSC based on pristine mesoporous TiO_2_, which showed that the time taken for electron injection from the perovskite layer to 2% Ce-doped mesoporous TiO_2_ was shorter. The enhanced electron extraction ability was probably due to the optimized morphology of mesoporous TiO_2_ and the decrease in the *V*_fb_ value, which further resulted in the increase in the PCE of the cell.

**Table 3:** TRPL data of perovskite films based on TiO_2_ and 2% Ce–TiO_2_ ETLs

Sample	*τ*_ave_ (ns)	*τ*_1_ (ns)	Amplitude of *τ*_1_ (%)	*τ*_2_ (ns)	Amplitude of *τ*_2_ (%)
TiO_2_	51.93	1.96	0.22	51.94	99.78
2% Ce–TiO_2_	37.40	2.13	0.49	37.41	99.51

We introduced Ce element as an effective doping agent into mesoporous TiO_2_ to increase the PCE of perovskite solar cells. It was shown that doping of mesoporous TiO_2_ with Ce improved the optoelectronic properties of the ETL owing to the optimized morphology of mesoporous TiO_2_ and the decreased *V*_fb_ value. The PCE of a cell increased to 17.75% in comparison with the PCE of an undoped device of 15.92%. The increases in the values of *J*_SC_ and *V*_OC_ are ascribed to the increase in the force of electron transport from the ETL to the FTO layer and the rise in the quasi-Fermi energy level. The increase in the FF is attributed to the increase in the value of *R*_rec_ after Ce was doped into the mesoporous TiO_2_ ETLs. This study shows that ion doping is an effective strategy for producing high-efficiency perovskite solar cells.

## Experimental section

### Materials and reagents

Chlorobenzene (99.9%), *N*,*N*-dimethylformamide (DMF, 99.9%), titanium isopropoxide (99.999%), and acetonitrile (99.9%) were obtained from Sigma-Aldrich. Cerium(iii) nitrate hexahydrate and isopropanol were obtained from Aldrich. 2,2′,7,7′-Tetrakis(*N*,*N*′-di-*p*-methoxyphenylamino)-9,9′-spirobifluorene (spiro-OMeTAD, 99.5%), PbI_2_ (99.99%), lithium bis(trifluoromethanesulfonyl)imide (Li-TFSI, 99.9%), CH_3_NH_3_I (99.5%), and 4-*tert*-butylpyridine (tBP, 96%) were obtained from Xi'an Polymer Light Technology Company. Glass substrates coated with fluorine-doped tin oxide (FTO) (15 Ω sq^−1^) were bought from OPV Tech New Energy Co. All the materials and reagents were used without further purification.

### Solar cell fabrication

The FTO substrates were ultrasonically cleaned with an abstergent, acetone, deionized water, and ethanol for 15 min in each case. The conductive substrates were dried with a nitrogen gun and post-treated with UV–ozone for 15 min. A c-TiO_2_ layer was coated on the cleaned FTO substrates by spin coating with the titanium precursor solution at 2000 rpm for 30 s. The as-prepared c-TiO_2_ was post-annealed at 150 °C for 15 min and then heated at 500 °C for 30 min in a muffle furnace. Then, we started to prepare the mesoporous TiO_2_ precursor solution, and 15 mL hydrochloric acid was mixed with 15 mL deionized water under magnetic stirring for 10 min. Then, 0.75 mL tetra-*n*-butyl titanate with or without different amounts of cerium(iii) nitrate pentahydrate (Ce(NO_3_)_3_·5H_2_O) as a dopant was added to the abovementioned mixed solution, and then the solution was vigorously stirred for 30 min to obtain a clarified solution. Afterwards, the clear solution was transferred into a reaction kettle equipped with compact TiO_2_/FTO substrates. The sealed reaction kettle was placed in a laboratory oven at 170 °C for 1 h. After the autoclave was cooled to room temperature naturally, the substrate was taken out, rinsed with deionized water and ethanol twice to remove residual reactants, and finally dried with a nitrogen gun and then annealed at 500 °C for 30 min in a muffle furnace. An MAPbI_3_ layer was spin-coated on c-TiO_2_/m-TiO_2_/FTO glass at 3000 rpm for 55 s. CB (80 μL) was washed onto the substrate during the spin-coating method about 10 s before the beginning of the procedure, and the substrate was then annealed at 100 °C for 20 min. An HTL film was manufactured by spin-coating a spiro-OMeTAD solution on a perovskite film at 3000 rpm for 30 s. Finally, an Ag electrode was deposited onto the spiro-OMeTAD layer by a sputtering technique to finish the manufacture of the cell.

### Characterization

The current–voltage characteristics of perovskite solar cells were determined using an electrochemical workstation under AM 1.5 simulated solar illumination (CEL-S500, Beijing, China). Mott–Schottky plots were recorded by employing an electrochemical workstation with a standard three-electrode configuration with Ag/AgCl as the reference electrode in saturated Na_2_SO_4_ and a Pt sheet as the counter electrode in deionized water. The morphology and composition of mesoporous TiO_2_ films were investigated using a scanning electron microscope (SEM; Zeiss EVOMA15) equipped with an energy-dispersive X-ray spectroscopy (EDX) detector. X-ray diffraction (XRD, DX-2700, Dandong) patterns were recorded from 22° to 58° with Cu Kα radiation (*λ* = 0.15406 nm) at a scanning rate of 4° min^−1^. X-ray photoelectron spectroscopy (XPS) measurements were carried out with an X-ray photoelectron spectrometer (Kratos Axis Ultra DLD) with a monochromated Al Kα X-ray source (*hν* = 1486.6 eV, 200 W). HRTEM images were recorded with a Hitachi HT-7700 transmission electron microscope (Hitachi Limited, Tokyo, Japan) at a voltage of 100 kV. UV-vis absorption spectra were recorded by a UV-vis spectrometer (Varian Cary 5000). Time-resolved photoluminescence (TRPL) spectra were recorded at 765 nm with an Edinburgh Instruments FLS 980 spectrometer with a pulsed diode laser at 485 nm (with an intensity of 0.12 mW cm^−2^) at a pulse frequency of 1 MHz. Incident photocurrent conversion efficiency (IPCE) spectra were recorded using an IPCE system (PVE 300, Bentham, Inc.) as a function of the wavelength from 300 to 800 nm. The active area of the perovskite solar cells was fixed at 0.16 cm^2^ using a mask.

## Conflicts of interest

There are no conflicts to declare.

## Supplementary Material

RA-009-C8RA07800A-s001
