# Supplementary material for: Crystal recombination control by using Ce doped in mesoporous TiO2 for efficient perovskite solar cells
Source: RSC Adv. 2019 Jan 9;9(2):1075–83. doi: 10.1039/c8ra07800a (PMC9059523; doi:10.1039/c8ra07800a)
Supplement: RA-009-C8RA07800A-s001 [file RA-009-C8RA07800A-s001.pdf]

Electronic Supplementary Material (ESI) for RSC Advances.
This journal is © The Royal Society of Chemistry 2018

Support information

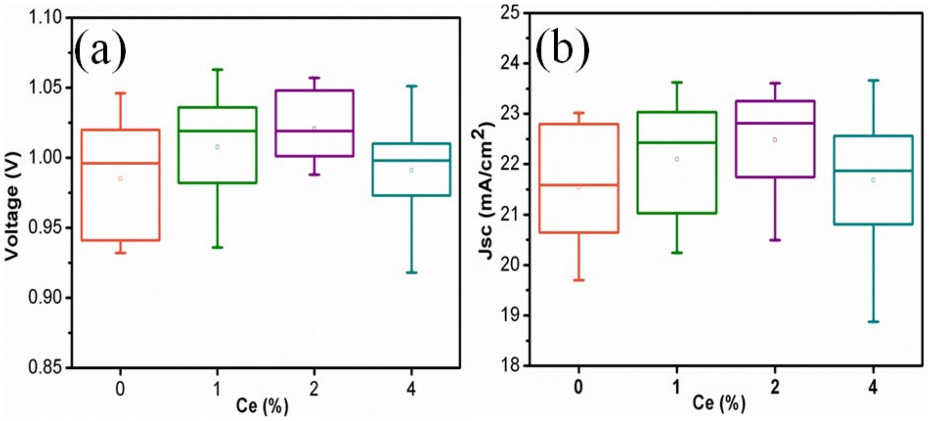

Figure. S1 (a and b) error bar of Voc, Jsc.

Table S1 Photovoltaic performance of PSCs based on TiO_2 and Ce-TiO_2 ETLs.

ETLs	V_{OC} (V)	J_{SC} (mA/cm^2)	FF (%)	PCE (%)
	Max (Average)			
TiO_2	1.020 (0.998)	22.80 (21.41)	68.50 (65.01)	15.92 (13.86)
1% Ce-TiO_2	1.042 (1.007)	23.62 (22.09)	67.60 (66.30)	16.63 (14.76)
2% Ce-TiO_2	1.048 (1.026)	23.61 (22.41)	71.70 (69.31)	17.75 (16.05)
4% Ce-TiO_2	1.005 (0.990)	22.56 (21.56)	69.50 (65.40)	15.74 (14.03)
